# Influence of Disinfection Methods on Cinematographic Film

**DOI:** 10.3390/ma16093493

**Published:** 2023-05-01

**Authors:** Vítězslav Knotek, Michal Ďurovič, Bohumil Dolenský, Zdeněk Hrdlička

**Affiliations:** 1 Department of Chemical Technology of Monument Conservation, University of Chemistry and Technology Prague, Technická 5, 166 28 Prague, Czech Republic; 2 Department of Analytical Chemistry, University of Chemistry and Technology Prague, Technická 5, 166 28 Prague, Czech Republic; 3 Department of Polymers, University of Chemistry and Technology Prague, Technická 5, 166 28 Prague, Czech Republic

**Keywords:** disinfection, cellulose triacetate, triphenyl phosphate, ^1^H NMR, ethylene oxide

## Abstract

Microbiological contamination of cinematographic films can cause damage and loss of image information. A large part of the films is made with the base of cellulose triacetate, which has been used from the 1940s until today. Cellulose triacetate is relatively resistant to common organic solvents, but some types of microorganisms can contribute to its faster degradation. In this work, we tested four types of disinfectants suitable for mass disinfection and sufficiently effective against various types of microorganisms. Butanol vapours, a commercial mixture of alcohols (Bacillol^®^ AF), Septonex^®^ (an aqueous solution of [1-(ethoxycarbonyl)pentadecyl] trimethylammonium bromide) and ethylene oxide applied as a gas mixed with carbon dioxide were tested. Samples of a commercial film made of cellulose triacetate were disinfected. The samples were aged for 56 days at 70 °C and 55% RH. Changes in optical, mechanical and chemical properties were studied. None of the disinfectants affected the change in the degree of substitution. For samples disinfected with Bacillol^®^ AF (alcohol mixture), part of the plasticiser (triphenyl phosphate) was extracted and the intrinsic viscosity of the cellulose triacetate solution was reduced after ageing. A slight decrease in intrinsic viscosity also occurred after disinfection with ethylene oxide. Compared to the non-disinfected samples, butanol vapours and Septonex^®^ appear to be the most gentle disinfectants for the cellulose triacetate film base, within the studied parameters.

## 1. Introduction

A cinematographic film is a complex material consisting of a polymer base on which an emulsion light-sensitive layer consisting of gelatine with a dispersed light-sensitive substance is deposited. The first film strip was made from cellulose nitrate in 1890 [1]. Due to its flammability and tendency to degrade, it was replaced by cellulose triacetate (CTA), which has been used since the 1940s [2]. In the 1960s, polyethene terephthalate (PET) was used for the first time as a cinematographic base [3]. Together with PET, the CTA base is still produced and used today. The main part of the collection of film material consists of material based on cellulose triacetate [4]. In comparison to the PET base, the disadvantage of the CTA base is its lower chemical stability and content of plasticisers. The CTA base always contains plasticisers that prevent dimensional changes with moisture fluctuations and in some cases serve as flame retardants [5]. There is also one advantage resulting from the lower tensile strength compared to a PET base and therefore less risk of damage to the projection equipment if the film gets stuck.

The conditions of both the film base and the emulsion layer are important for the preservation of audiovisual information. Inappropriate storage conditions promote the chemical degradation of the base and the growth of microorganisms. The emulsion layer is more susceptible to microbial attack due to the presence of gelatine [4,6]. Moulds, in particular, are capable of causing extensive damage by growing hyphae through the gelatine emulsion layer, causing irreversible damage to the emulsion layer and may also cause separation from the substrate [3]. In the case of a microbiological attack on the emulsion layer, the polymer substrate is also attacked [7]. Cellulose acetate has been intensively investigated for its potential use as a biodegradable plastic [8]. The resistance of cellulose acetate was found to be related to the degree of substitution (DS). The higher the degree of substitution, the higher the resistance to biodeterioration [9,10]. The CTA used as the film base has DS greater than 2.7. However, the DS of the film is reduced by various mechanisms when stored at higher temperatures and humidity [11]. Degraded cinematographic films must be separated and the degradation rate reduced by freezing [1]. In the event of a proven microbiological attack, some disinfection method should be used, because handling the attacked material poses a health risk to curators and restorers.

A wide range of chemical substances can be used for disinfection purposes [12]. Alcohols, aldehydes, ethylene oxide, quaternary ammonium ions, phenols, and others are recommended for the disinfection of objects of cultural heritage [13]. When choosing a suitable disinfectant, the effectiveness against individual types of microorganisms should be considered. The determination of microorganisms is carried out using a suitable smear technique and microbiological testing [14]. Alcohol is used very often and for a long time. To ensure a sufficient disinfecting effect, a certain minimum concentration of alcohol and a certain endurance time is required [15]. However, bacterial spores tend to be resistant to alcohol [16,17]. Ethylene oxide is more effective in this regard [18].

In the last two decades, many articles have dealt with the identification of microbiological settlements for cinematographic films and their influence on degradation [4,7,14,19,20,21,22,23,24,25]. Finding the presence of harmful microorganisms in cinematographic films always presents a problem in the way of removing these microorganisms [14]. The recommended procedures to remove microorganisms from contaminated films are limited to the use of cleaning solutions [26] or mainly toxic substances [3,27]. Moreover, there is a lack of comprehensive research on the influence of recommended substances on cinematographic films. We found only one current article dealing with the effect of a physical method of disinfection on cinematographic films. [28]. Since the majority of cinematographic films in the archives were produced on a triacetate base, this work investigates the influence of four common disinfection methods on the properties of the triacetate (CTA) base.

## 2. Materials and Methods

### 2.1. Tested Materials

A commercial film base made of cellulose triacetate (CTA, FOMA Bohemia, Czech Republic) was tested. A thin layer of gelatine (the so-called subbing layer) was present on one side of the base. The film base was cut into samples for disinfection and measurement purposes. After disinfection, the samples were artificially aged in a chamber (Memmert HCP 108) at 70 °C and 55% RH for 56 days. The RH value was chosen according to ISO 18911:2000. Labelling of samples after disinfection is shown in Table 1. The abbreviations of Table 1 are used for the identification of tested samples in Section 3 (Results and Discussion).

### 2.2. Disinfection Methods

Disinfection methods were selected from among the most widely used methods in the field of conservation and restoration of cultural heritage objects, taking into account the proven effectiveness against microorganisms [29]. Furthermore, only methods suitable for mass disinfection of specific materials such as film strips were selected. Two representatives were selected from the group of alcohols: butanol vapours and a commercial mixture of alcohols Bacillol^®^ AF (BODE Chemie GmbH, Hamburg, Germany). Ethylene oxide was chosen from the group of epoxides. A solution of [1-(ethoxycarbonyl)pentadecyl] trimethylammonium bromide (known as Septonex^®^ (Herbacos Recordati s.r.o., Pardubice, Czech Republic)) was chosen as a representative of quaternary ammonium salts.

#### 2.2.1. Butanol Vapours

An aqueous solution of n-butanol with a concentration of 96 wt.% was prepared by mixing n-butanol (99%, Ing. Petr Švec—PENTA s.r.o., Prague, Czech Republic) with deionised water. The samples were placed in a plastic desiccator. A Petri dish with a 96% n-butanol solution was placed at the bottom of the desiccator. The amount of solution in the Petri dish was determined based on the ratio of 100 mL of the solution to the volume of 30 dm^3^ of the disinfection chamber. Disinfection was carried out for 48 h at 23 ± 2 °C.

#### 2.2.2. Bacillol^®^ AF

Disinfection with Bacillol^®^ AF (Bode Chemie, Hamburg, Germany) was carried out by immersing the samples in an undiluted Bacillol^®^ AF disinfectant. The active ingredients of Bacillol^®^ AF are alcohols (propan-1-ol, propan-2-ol and ethanol). Exposure was carried out for 5 min at 23 ± 2 °C. The drying process took place by leaving the samples free in the air.

#### 2.2.3. Septonex^®^

Septonex^®^ is an aqueous solution (2% *w*/*v*) of [1-(ethoxycarbonyl)pentadecyl] trimethylammonium bromide (Dr. Kulich Pharma s.r.o, Hradec Králové, Czech Republic), a quaternary ammonium salt with disinfectant effect. The film samples were immersed in Septonex^®^ for 1 min. Subsequently, the samples were immersed for 1 min in deionised water and dried between sheets of filter paper, separated by a layer of non-woven polyester fabric.

#### 2.2.4. Ethylene Oxide

A mixture of ethylene oxide and carbon dioxide gases (10% ethylene oxide + 90% CO_2_) was used for disinfection. The samples were stored in the MATACHANA (Antonio Matachana, S.A., Barcelona, Spain) disinfection chamber (type 1.3100 LGE-2) at the National Archives Czech Republic. The disinfection took place for 6 h at 30 ℃ and a pressure of 220 kPa. After the disinfection, the samples were ventilated for 6 days in a ventilation tunnel with air heated to 30 °C. Subsequently, the samples were transferred to a special chamber where the concentration of ethylene oxide was monitored by GC-MS for 24 h.

### 2.3. Colorimetry

The colour of the films was measured with a CM-700d spectrophotometer (Konica Minolta, Tokyo, Japan) in the CIE L*a*b* colour space [30] using a colour-stable white pad. The spectrophotometer was controlled using the SpectraMagic NX programme. The total colour difference ΔE* was calculated according to Equation (1) [30], where $L_{0}^{*}$, $a_{0}^{*}$, $b_{0}^{*}$ are the colour parameters before disinfection and before artificial ageing and $L_{1}^{*}$, $a_{1}^{*}$, $b_{1}^{*}$ are the colour parameters of the disinfected samples after artificial ageing.

(1) $$\Delta E^{*}=\sqrt{{\left(L_{1}^{*}-L_{0}^{*}\right)}^{2}+{\left(a_{1}^{*}-a_{0}^{*}\right)}^{2}+{\left(b_{1}^{*}-b_{0}^{*}\right)}^{2}}$$

Ten measurements were evaluated on each side of each sample. The average value and standard deviation of ten values for each sample set were evaluated.

### 2.4. Gloss Measurement

The gloss was measured by a micro-TRI-gloss instrument (BYK Additives & Instruments, Wesel, Germany) at ten locations on each side of the sample. Since the film base shows high gloss, the 20° geometry was chosen for the measurements. The average value and the absolute standard deviation were evaluated from ten values for each sample set.

### 2.5. Mechanical Properties

The testing was performed on a universal testing system Instron 3365 (Instron, Norwood, MA, USA). Measurements were carried out according to the ISO 18901:2010(E) standard. Ten measurements were made for each set of samples. The tensile strength and elongation at the break of the samples were evaluated. The average value and standard deviation were calculated for both parameters.

### 2.6. Attenuated Total Reflectance-Fourier Transformed Infrared Spectroscopy (ATR-FTIR)

To determine the type of plasticiser used in the production of cellulose acetate, the film was extracted with methanol according to the procedure described in the literature [31]. After evaporation of the methanol, the extract was analysed using an ATR-FTIR spectrometer (Nicolet iZ10, MCT detector, diamond crystal, resolution 4 cm^−1^, 64 scans). The extracted plasticiser was identified as triphenyl phosphate (TPP).

**Figure 1:**
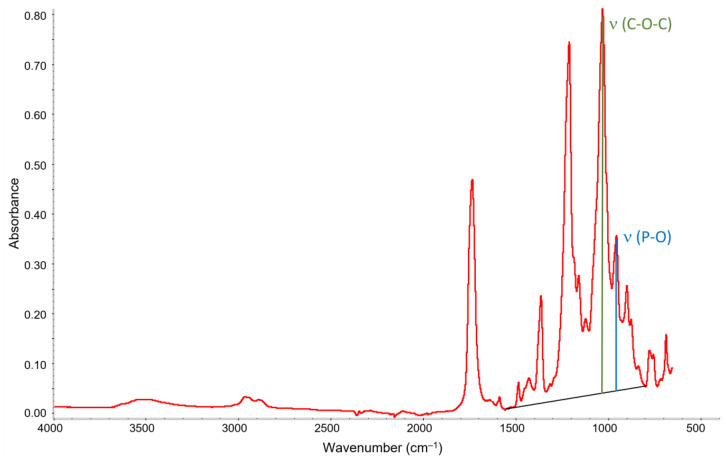
FTIR spectrum of the triacetate base with marked bands was used to calculate the relative plasticiser content.

ATR-FTIR spectra were used to compare the plasticiser content in the surface layer of the samples. For comparison, the absorbance intensity ratio was determined for the bands corresponding to the vibrations of the bond (P-O) belonging to the plasticiser and the bond (C-O-C) belonging to cellulose acetate. The determination of the observed peak intensities is shown in Figure 1. No baseline correction was applied to the spectra. Only the local baseline between 1560 and 800 cm^−1^ was used to classify the height of the bands. This method was adapted from [2]. Average values were determined from 10 measurements for each sample. Subsequently, the standard deviations were evaluated.

### 2.7. NMR Spectroscopy

The NMR spectra were recorded by a 500 MHz instrument (JEOL ECZ 500R, Tokyo, Japan). Chemical shifts (δ) are presented in ppm and coupling constants (*J*) are presented in Hz. The ^1^H and ^13^C chemical shifts are referenced to tetramethylsilane using the solvent signals CHD_2_SOCD_3_ (2.50 ppm) and CD_3_SOCD_3_ (39.52 ppm).

#### 2.7.1. Preparation of Samples for NMR Analyses

For the preparation of NMR samples, the gelatine subbing layer was removed from the surface of each film sample using a scalpel. Approximately 15 mg of a film sample and optionally approximately 5 mg of 1,2,4,5-tetrachloro-3-nitrobenzene as the internal standard (IS, the certified reference material of 99.74% purity, Supelco Inc., Bellefonte, PA, USA) were accurately weighed (an ultra-micro balance, Sartorius Cubis II, Germany, tolerance 0.20 μg) into a vial, 600 μL of the DMSO-*d*_6_ (99.80% D, a standard quality NMR solvent, Eurisotope, Saint-Aubin, France) was added and the vial capped. The vial was slowly rotated several times to mix its content. A transparent solution was obtained after 1–2 days. Then 500 μL of the viscous solution were transferred into the standard 5 mm NMR Tube (WG-1000-7, Wildman). Three samples were prepared from each film sample.

#### 2.7.2. Measurement and Processing of Quantitative ^1^H NMR Spectra (qNMR)

Each sample was loaded into the NMR spectroscope and heated to 95 °C. After 20 min, the sample was locked and shimmed via gradient shimming followed by the long autoshim procedure on Z1 to Z4 axes. Then the qNMR spectrum was recorded using a nutation angle of 45° (3.25 μs), a spectral offset of 6 ppm, spectral width of 19 ppm, an acquisition time of 4 s, a digital resolution of 0.25 Hz, a relaxation delay of 60 s, no sample spinning, ^13^C decoupling MPF8, and 256 scans. The total measurement time was 5 h, including the sample temper and the shimming. The recorded FID was fulfilled to 512 k of points and processed by the common Fourier transformation. The spectrum was phased manually and the baseline correction by a polynomial fit of order 1 was applied, then referenced. Since the signals of the spectrum had mostly non-ideal shapes, the standard running sum method was used for integration [32].

#### 2.7.3. Identification of Glucose Units

For the elaboration of the CTA molecular structure, we recorded the standard 2D correlation NMR experiments such as DQF-COSY, HSQC, HMBC, TOCSY and NOESY at 120 °C.

#### 2.7.4. Estimation of Average Degree of Substitution

The degree of substitution (DS) was calculated using Equation (2) [33], where *I_Me_* represents the integral intensity for the methyl groups (2.4605 to 1.3561 ppm). *I_Glu_* represents the integral intensity for the signals of glucose units (6.0078 to 3.0679 ppm), however, also including the signals of unsubstituted OH groups (see the discussion). Therefore, an iterative calculation was performed to correct the signal of the OH groups to obtain real DS. In Equation (2), *DS*_*n*−1_. is equal to 3 for the first iteration.

(2) $$DS_{n}=\frac{I_{Me}}{I_{Glu}}\cdot \frac{7+\left(\frac{3-DS_{n-1}}{3}\right)}{3}$$

#### 2.7.5. Triphenyl Phosphate Quantification

To determine the plasticiser content, it was necessary to determine the moisture content in the examined films. Moisture content was determined using a moisture analyser (KERN DBS60-3, Germany). Approximately 0.7 g of foil was dried at 105 °C for 15 min.

The content of triphenyl phosphate (*C_TPP_*) was calculated using Equation (3) [32], where *M_IS_* and *M_TPP_* are the molar weights of IS and TPP respectively, *P_IS_* is the purity of IS, *P_TPP_* is a dry matter of the *CTA* sample, *I_IS_* is the integral of *IS* (8.3795 to 8.2925 ppm), *I_TPP_* is the integral of *TPP* (7.5273 to 7.1982 ppm), *N_IS_* is 1, *N_TPP_* is 15, *m_IS_*, and *m_CTA_* are weights of *IS* and *CTA*.

(3) $$C_{TPP}\;\left(\mathrm{wt}.\%\right)=\frac{M_{TPP}}{M_{IS}}\cdot \frac{I_{TPP}}{I_{IS}}\cdot \frac{N_{IS}}{N_{TPP}}\cdot \frac{P_{IS}}{P_{TPP}}\cdot \frac{m_{IS}}{m_{CTA}}$$

### 2.8. Intrinsic Viscosity

Measurements were carried out according to the technical standard ASTM D871-96 (2019). Before dissolution, the gelatine subbing layer was removed with a scalpel and the plasticiser was completely extracted with methanol according to the literature [21]. Approximately 0.26 g of sample were dissolved in 100 mL of a solvent mixture of dichloromethane and methanol (90:10 by weight). An Ubbelohde viscometer (diameter 0.4 mm, K = 0.003001) was used for the measurement at 25 °C. The intrinsic viscosity value was calculated according to Equation (4), where [*η*] is the measured intrinsic viscosity, *k* is the solvent constant (in this case *k* = 3), *c* is the polymer concentration in g/dL, *η_rel_* is the relative viscosity calculated as the ratio of the average solvent flow time to the average polymer solution flow time. The calculation of intrinsic viscosity is based on the modified Baker-Philippoff equation [34]. Two samples were prepared and measured for each set of samples. Each measurement was repeated at least once.

(4) $$\left[\eta \right]=\frac{k}{c}\cdot \left(10^{\frac{\log\eta_{rel}}{k}}-1\right)$$

## 3. Results and Discussion

### 3.1. Colorimetry

**Figure 2:**
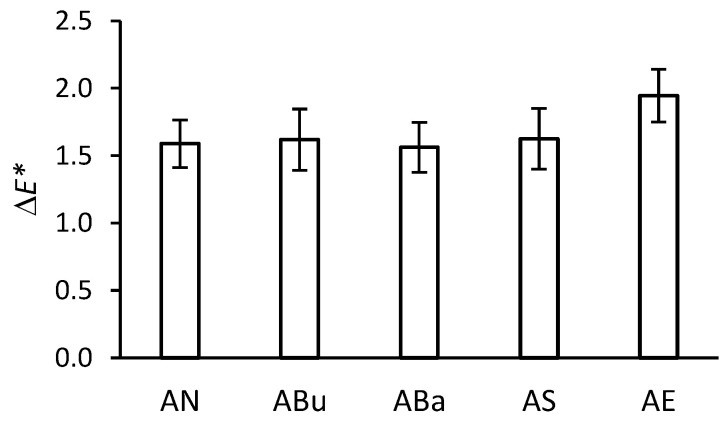
Total colour difference (ΔE*) of the acetate side of the samples after disinfection and artificial ageing.

The stability against the colour change of the base is a very important parameter for cinematographic films, as it affects the colour of the resulting image during projection. The total colour difference for the acetate side of the samples (without the gelatine subbing layer) is shown in Figure 2. Except for the samples disinfected with ethylene oxide (AE), the total colour difference is similar to the non-disinfected samples (AN). The higher value of ΔE* for the AE samples is primarily due to a shift along the L* coordinate toward lower values, i.e., the substrate has darkened slightly. As a result of ethylene oxide disinfection, there was no yellowing, which is typical of polymer oxidation [35,36]. Therefore, it can be concluded that the change in colour after disinfection with ethylene oxide and ageing was not caused by oxidation.

**Figure 3:**
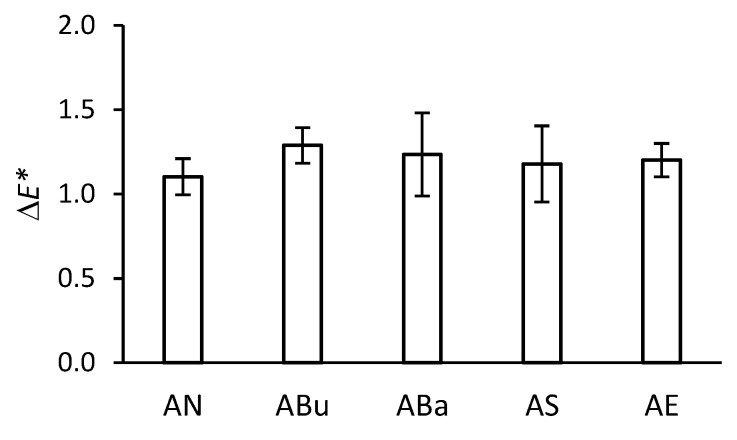
Total colour difference *(*ΔE*) of the side with the gelatine subbing layer after disinfection and artificial ageing.

The total colour difference of the side with the gelatine subbing layer is shown in Figure 3. The results are very similar for individual disinfection methods. The ABu samples showed the highest colour change. Compared to the non-disinfected samples, there was a shift along the L* coordinate to positive values, i.e., a slight lightening of the surface with the gelatine subbing layer. No yellowing of the gelatine subbing layer was detected.

### 3.2. Gloss Measurement

**Figure 4:**
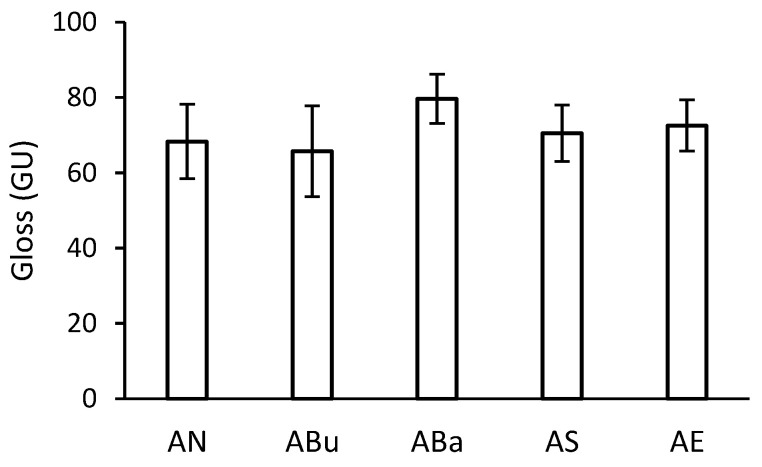
Gloss of disinfected samples after artificial ageing.

Gloss depends on the condition of the surface, especially the flatness of the surface [37]. After disinfection, there was no deformation of the samples that could affect the gloss measurement. The gloss of the acetate side is shown in Figure 4. The highest gloss was measured for the ABa samples. The average gloss values were approximately 10 gloss units higher than those of the other samples. The increase in gloss for the ABa samples is most probably related to the effect of the amount of plasticiser in the substrate (see Section 3.4 and Section 3.5).

### 3.3. Mechanical Properties

**Figure 5:**
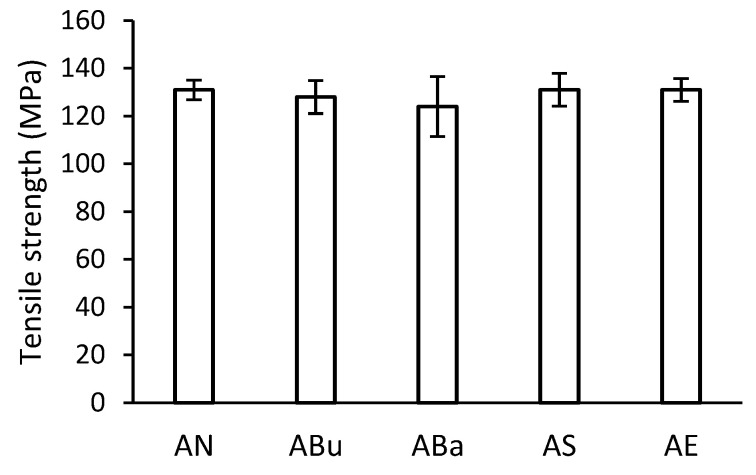
Tensile strength of disinfected samples after artificial ageing.

The tensile strength of the disinfected samples after ageing is shown in Figure 5. The results do not show a significant effect of disinfection on the change in the strength of the samples. However, when the mean tensile strength values are compared, the lowest tensile strength can be seen for the ABa samples. In general, tensile strength depends on several factors, e.g., an average degree of polymerisation, plasticiser content, crystalline phase content, the conformation of macromolecules, and others.

**Figure 6:**
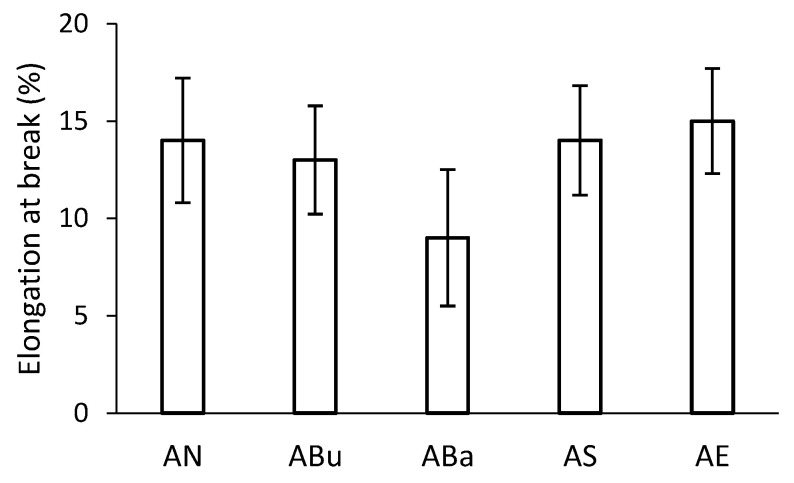
Elongation at break of disinfected samples after artificial ageing.

Larger differences can be observed in the case of elongation for aged samples (Figure 6). Elongation is related to both the average degree of polymerisation and the plasticiser content. When comparing the results in Figure 6, there was a significant decrease in elongation for the ABa samples. Furthermore, the ABa samples showed a high variance of values, which was reflected in the size of the error bars. This may indicate an inhomogeneous composition of the ABa samples after disinfection.

### 3.4. ATR-FTIR Spectroscopy

**Figure 7:**
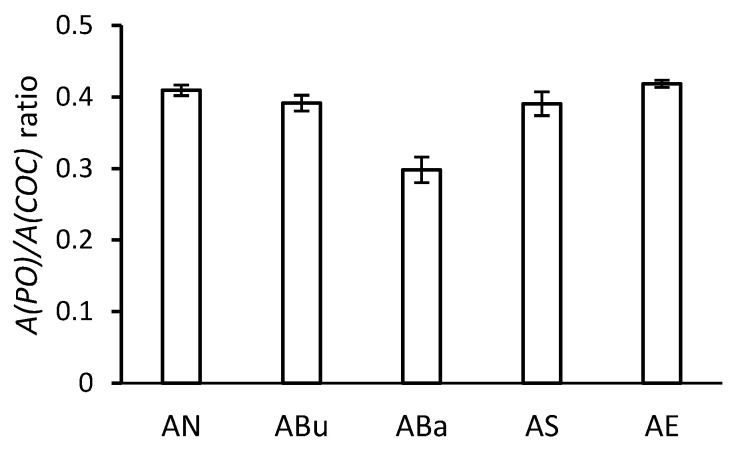
Height ratios of selected bands in IR spectra to determine the plasticiser content of the samples.

The results of the band ratio of the ATR-FTIR spectra of the disinfected and aged samples are shown in Figure 7. The values obtained by the ATR-FTIR method using a diamond crystal express the composition of the base at a depth of several micrometres. Figure 7 shows the relative content of triphenyl phosphate (TPP) in the thin layer below the surface of the samples. The ABa samples show the lowest content of TPP. The Bacillol^®^ AF disinfectant contains ethanol and propanol isomers. After 5 min of contact with Bacillol^®^ AF with the base, some of the TPP was extracted from the samples. The lower plasticiser content was reflected in the reduced elongation of the ABa samples (see Figure 6). The increase in the gloss of the ABa samples after ageing may also be related to the lower plasticiser content (see Figure 4).

It can also be seen in Figure 7 that compared to the untreated AN samples, the ABu and AS samples had a slightly reduced TPP content ratio. Interestingly, the elongation of the ABu and AS samples (see Figure 6) is slightly lower compared to the AN samples without disinfection. During disinfection, the AS samples were immersed in the aqueous solution of a quaternary ammonium salt. This salt can serve as both a disinfectant and a surfactant. A small amount of TPP could be extracted from the surface layers of the film as a result of the reduction in the surface tension between water and TPP. In contrast, the ABu samples were exposed to n-butanol vapours, which can at least superficially enter the base structure and interact with TPP. As a result of the presence of n-butanol in the surface layers of the foil, TPP can diffuse to the surface and its concentration can increase locally after the n-butanol evaporates. During artificial ageing, diffusion of TPP back into the film or release into the environment may occur.

For the AE samples, the TPP content is comparable to that of the AN samples. Therefore, we assume that ethylene oxide disinfection does not affect the reduction of the TPP content of the base.

### 3.5. NMR Spectroscopy

To the best of our knowledge, this is the first case of triphenyl phosphate (TPP) quantification in film samples using ^1^H NMR spectroscopy, therefore, we follow the general recommendations to record qNMR spectra [32,38].

Firstly, we explored the NMR characteristics of a film sample. Both ^1^H and ^13^C NMR signals of TPP and internal standard (IS) were sharp, however, all signals of CTA were too broad to observe their multiplicity or to set reasonable integration limits at room temperature. A diluted sample resulted in narrower signals in the spectrum, however, the SNR decreased significantly, which would require excessive time to record qNMR spectra. Therefore, we measured the spectra at elevated temperatures (from 22 °C to 148 °C), which significantly narrowed the signals (Figures S1–S3).

The maximum effect of temperature was reached at approximately 120 °C, where the signals are so narrow that their basic multiplicity and signals of minor species were recognised. Based on the analysis of 2D NMR (the AS sample was used), we identified four different glucose units in a molar ratio of approximately 80:10:9:1. Although we were unable to assign all ^1^H and ^13^C signals and correlations for each unit due to low-intensity and signals overlaps, we were able to identify their structure based on the marvellous works of Kono et al. [39] and Hikichi et al. [40]. For the comparison of the chemical shifts, see Tables S1 and S2.

The main glucose unit was identified as the 2,3,6-triacetylated glucose unit based on our assignment of all ^1^H and ^13^C signals and the agreement of the chemical shifts with the literature [39,40].

The observed ^1^H and ^13^C signals from the second glucose unit are in accordance with those of 2,3-diacetylated glucose units [39].

The third glucose unit is characterised by the signals of the chemical shifts 4.746 (d, H1), 4.599 (t, H2), 5.054 (t, H3), and 3.758 (t, H4), which are not mentioned by Kono [39], although our inspection of their published 2D TOCSY NMR spectrum revealed that the signals were presented. Since the signals are very similar to those of the main 2,3,6-triacetylated glucose unit, we agree with the conclusions of Hikichi [40] that the chemical shifts of a glucose unit are affected by the surrounding units within the polymer backbone. Since the Hikichi sample has a degree of substitution (DS) of only 2.46 they observed even four different 2,3,6-triacetylated glucose units [40]. Since our sample has a much higher DS (2.85) we observed only two different 2,3,6-triacetylated glucose units. The major one is surrounded by 2,3,6-triacetylated glucose units, and the minor one is surrounded by one 2,3,6-triacetylated and one 2,3-diacetylated glucose unit. The 9% content of the different 2,3,6-triacetylated glucose units is consistent with the 10% content of the identified 2,3-diacetylated glucose unit (vide supra); therefore, the signals of its second 2,3,6-triacetylated glucose neighbour are very similar to the major one. The option that the 2,3-diacetylated unit is mainly at the end of the polymer chain is improbable due to the high degree of polymerisation [41] (if the degree of polymerisation is 300 to 1700, there is less than 0.7% of the ending units). 

The fourth glucose unit is characterised by the signals of the chemical shifts 4.367, 3.155, 4.833, 3.576, 3.619 and 4.528, which are in agreement with those published for H1, H2, H3, H4, H5 and H6a, respectively, of the 3,6-diacetylated glucose unit [39].

Unfortunately, the high temperature complicates the use of an automotive gradient shimming due to the higher thermal convection within the sample. In addition, the water content can be a source of other problems at a temperature above its boiling point, especially during long-time qNMR experiments. Therefore, we decided to record the qNMR spectra at 95 °C. For three various samples, we also measured the spectra (8 scans) before and after the qNMR experiments. Since the spectra were identical, we concluded that the sample is stable under the chosen condition. It should be noted that the signal of water is narrow (HWHH = 3.5 Hz) for a fresh sample, but broad (HWHH = 40.0 Hz) after a few weeks, for which we have no experimentally supported explanation. We also recorded the ^1^H NMR spectrum of the gelatine subbing layer, which contained particularly the signals of CTA, therefore, the layer probably does not significantly affect the quantification (Figure S4).

To estimate the proper parameters to record the qNMR spectra, we estimated relaxation time T_1_ using the standard inversion recovery experiment processed by the JEOL Delta 5.3.1 software of the NMR spectroscope. Since such estimation is time-consuming, we studied only three samples with almost identical values. By the standard nutation experiment, we estimated the 90° x-pulse duration is 6.50 μs. Then we found that the T_1_ value is 13.0 s for IS, 6.6 to 9.0 s for TPP signals, 1.6 to 1.8 s for the glucose signals, and 1.9–2.0 s for signals of CTA methyls. Therefore, to record qNMR spectra in reasonable time we used a 45° nutation pulse and a repetition time of 64 s. As we recorded qNMR spectra with 256 scans, we acquired spectra with a sufficient signal-to-noise ratio (SNR) of approximately 14,000 for IS signal, 1600–4000 for TPP signals, 240–2700 for the glucose signals, and 750 to 23,000 for signals of methyl groups.

**Figure 8:**
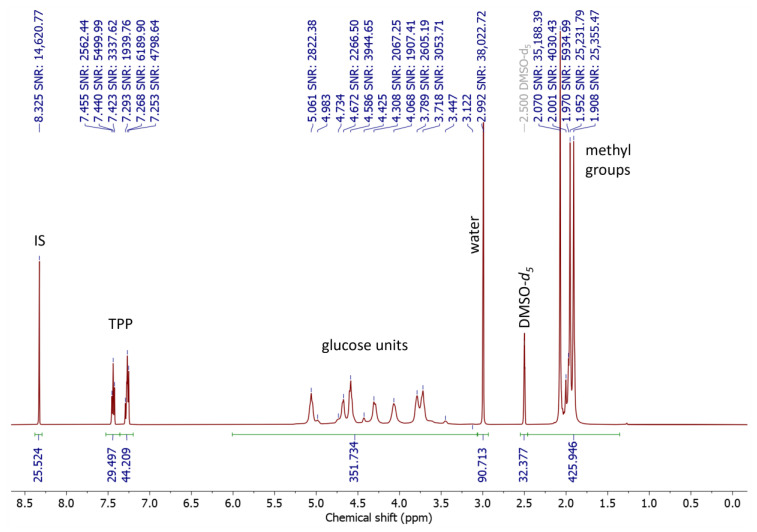
^1^H NMR spectrum of the CTA sample (AS) with added IS after peak area integration.

The integration range for qNMR is required to be 20 times the HWHH of the peak of interest. This was fulfilled for the IS signal, which is a well-isolated singlet. The signals of TPP have multiplicity and are close to each other. Thus, the limits were set from the most left and the most right signals of the multiplets, and then, divided at the middle (see Figure 8), hence, 7.5273 to 7.3581 ppm for the meta hydrogen and 7.3577 to 7.1982 ppm for the ortho and para hydrogen were used. It should be noted that the ratio of the TPP integrals on all samples was 1.495 to 1.502, which confirms that the integration was accurate; their sum was used for calculations (see Table S3). Unfortunately, the signals of the glucose units as well as the methyl groups were too broad to fulfil the general qNMR requirement. Hence, we estimated the integral range manually by enlarging the range until the change in the integral value was negligible.

To estimate the DS of CTA we tested the recently described method based on the recording of the ^13^C qNMR spectrum of a CTA sample without any chemical pretreatment [39]. Unfortunately, even after 15 h of measurement, we obtained a ^13^C NMR spectrum with signals of SNR approximately 2, which is useless for quantification (see Figure S5); a much more concentrated sample or a sensitive spectroscope would be required.

Then we calculated the DS on the basis of the identified glucose units (vide supra). Although the signals are not separated enough to be accurately integrated, we obtained a reasonable value of DS 2.87, which should be understood as the highest possible value, since only four major species were identified and taken into the calculation.

Finally, we calculated the DS using the ratio of the integral intensities of the glucose unit signals and the methyl signals. However, the integral area of the glucose unit signals also includes the signals of the unsubstituted OH groups, as we confirmed by recording the ROESY1D spectrum with the water signal irradiation (mix time 250 ms), in which a broad signal at 4.45 ppm is observed as a consequence of the chemical exchange between OH and water. Thus, the integral of the glucose signals has to be normalised by dividing by seven (the number of CH glucose unit hydrogen atoms) plus the molar content of the unsubstituted OH group, which is, however, unknown. Thus, we used the iterative approach (see Equation (2)). At first, we used normalisation by seven, then used the obtained DS to correct the normalisation and get a more accurate DS. Three iterations were sufficient to obtain the final DS values (see Table 2 and Table S3).

The results of the DS and TPP content for disinfected and aged samples are summarised in Table 2. The NMR data show that disinfection does not affect the degradation by decreasing the DS. All samples showed a DS of around 2.85. However, some changes can be found in the TPP content. Compared to the AN samples, the ABa samples contained approximately 1% less TPP. For the other samples (ABu, AS, and AE), the TPP content differs negligibly and is very similar to that of AN. The results confirmed the ATR-FTIR measurements, where the ABa samples contained the relatively lowest amount of TPP.

### 3.6. Intrinsic Viscosity

The intrinsic viscosity of the cellulose triacetate solution could be related to the average degree of polymerisation. A role can also play in the size and shape of the CTA macromolecules in the solution. The intrinsic viscosity number can be used to estimate the molecular weight of cellulose triacetate by using the Mark-Houwink equation using proper constants for the specific solvent-temperature-polymer system. In general, the higher the intrinsic viscosity, the higher the average degree of polymerisation. However, gel permeation chromatography (GPC) analysis would confirm this general assumption.

**Figure 9:**
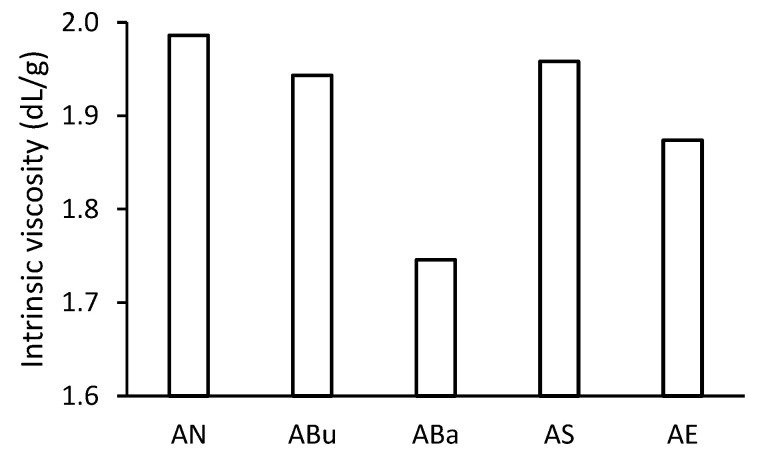
The intrinsic viscosity of solutions of samples after artificial ageing.

All disinfected samples showed a decrease in intrinsic viscosity compared to the non-disinfected AN samples (Figure 9). The ABa samples showed the lowest intrinsic viscosity value. The lower average degree of polymerisation of the ABa samples supports the result of the tensile strength measurement (see Figure 5). For the ABa samples, the reduction in the degree of polymerisation may be related to the reduced content of the plasticiser [41]. The structure of CTA without the plasticiser is more accessible to water vapours. In addition to UV radiation, water (specifically H_3_O^+^) can cause heterolytic cleavage of the main cellulosic chain [2]. Another possibility may be the attack of the glycosidic bond by the alcohols contained in the Bacillol^®^ AF disinfectant and the cleavage of the bonds by the alcoholysis mechanism [42]. The ABu and AS samples showed a slight decrease in intrinsic viscosity (see Figure 9). The slightly lower TPP content of these samples (see Figure 7) would support the association of plasticiser content with an average degree of polymerisation of CTA after ageing. However, the intrinsic viscosity also decreased for the AE samples, where the plasticiser content was at the level of the non-disinfected AN samples. In this case, the reaction of ethylene oxide with a glycosidic bond comes into consideration. Some studies indicate a slight decrease in the average degree of polymerisation for paper, but the mechanism of the reaction is still unclear [43].

## 4. Conclusions

In this paper, a method for accurate measurement of the content of triphenyl phosphate in cellulose acetate was developed using the ^1^H NMR technique. The results correlated well with the trends found using the non-destructive FTIR method. Furthermore, the degree of substitution of cellulose triacetate after disinfection was measured very precisely using the ^1^H NMR technique, and it was found that the disinfection method has no effect on the change in the degree of substitution.

Bacillol^®^ AF cannot be recommended for the disinfection of the CTA film base, because after its use there was a decrease in mechanical properties, a significant decrease in the content of plasticiser, and a decrease in the intrinsic viscosity value. A slight decrease in intrinsic viscosity compared to the results after the use of butanol vapours and Septonex^®^ products also occurred after disinfection with ethylene oxide. However, the mechanism of action of the disinfectant on the cleavage of the glycosidic bond could not be reliably clarified. In the other parameters compared, the results for ethylene oxide were comparable to those of butanol vapours and Septonex^®^ disinfectants. Butanol vapours and Septonex^®^ appear to be the most suitable disinfectants for a cellulose triacetate cinematographic base plasticised with triphenyl phosphate. The disinfected samples were close to the non-disinfected samples in terms of optical and mechanical properties, as well as the plasticiser content and intrinsic viscosity value. Further research will focus on the influence of disinfectants on real black-and-white cinematographic films with a sensitive emulsion layer.

## Figures and Tables

**Table 1:** Labelling of samples after disinfection.

Disinfection Method	Without Disinfection	Butanol Vapours	Bacillol^®^ AF	Septonex^®^	Ethylene Oxide
Label	AN	ABu	ABa	AS	AE

**Table 2:** DS and TPP content of disinfected and aged samples measured by NMR.

	AN	ABu	ABa	AS	AE
DS	2.85	2.84	2.85	2.85	2.84
TPP content (%)	10.50 ± 0.03	10.41 ± 0.03	9.49 ± 0.02	10.52 ± 0.01	10.54 ± 0.08

## Data Availability

Not applicable.

## Supplementary Materials

The following supporting information can be downloaded at: https://www.mdpi.com/article/10.3390/ma16093493/s1, Figure S1: Temperature dependence of ^1^H NMR spectra of the CTA sample (AS); Figure S2: Temperature dependence of ^1^H NMR spectra of the CTA sample (AS); comparison of the spectra at 22 °C (upper) and the best resolved ^1^H NMR spectrum (6.18 mg in 0.55 mL DMSO-*d*_6_ at 134 °C); a shimming deficiency was reduced by the reference deconvolution using the signal of IS; Figure S3: Temperature dependence of ^1^H NMR spectra of the CTA sample (AS) from 22 to 148 °C; the intensity of DMSO-*d*_6_ signal is set to be equal; Figure S4: Comparison of ^1^H NMR spectra of the gelatine subbing layer (blue) and the CTA sample (AE) (red); the intensity of glucose units is set to be equal; Figure S5: The ^13^C NMR spectrum of CTA in DMSO-*d*_6_ at 60 °C; Table S1: The chemical shifts of ^1^H NMR (ppm) relatively to TMS; Table S2: The chemical shifts of ^13^C NMR (ppm) relatively to TMS; Table S3: Data for calculation of DS and TPP content of CTA after disinfection.
