# Supplementary material for: Influence of Disinfection Methods on Cinematographic Film
Source: Materials (Basel). 2023 May 1;16(9):3493. doi: 10.3390/ma16093493 (PMC10180128; doi:10.3390/ma16093493)
Supplement: Supplementary file 1 [file materials-16-03493-s001.zip › Table S1.docx]

| **Table S1:** The chemical shifts of ^1^H NMR (ppm) relatively to TMS | | | | | | | |
| --- | --- | --- | --- | --- | --- | --- | --- |
| DMSO-*d*_6_, 90°C [Kono et al., 2015] | | |  |  |  |  |  |
| **Type of glucose unit** | **H1** | **H2** | **H3** | **H4** | **H5** | **H6a** | **H6b** |
| 2,3,6-triacetylated | 4.67 | 4.58 | 5.07 | 3.72 | 3.75 | 4.28 | 4.07 |
| 2,3-diacetylated | 4.62 | 4.55 | 4.96 | 3.78 | 3.41 | 3.76 | 3.62 |
| 2,6-diacetylated | 4.76 | 4.68 | 3.51 | 3.52 | 3.73 | 4.20 | 4.03 |
| 3,6-diacetylated | 4.35 | 3.12 | 4.83 | 3.58 | 3.64 | 4.38 | 4.07 |
| 2-acetylated | 4.70 | 4.62 | 3.48 | 3.53 | 3.44 | 3.67 | 3.58 |
| 3-acetylated | 4.48 | 3.27 | 4.95 | 3.62 | 3.40 | 3.82 | 3.62 |
| 6-acetylated | 4.36 | 2.98 | 3.40 | 3.39 | 3.76 | 4.29 | 4.00 |
| unsubstituted | 4.42 | 3.13 | 3.43 | 3.41 | 3.46 | 3.74 | 3.65 |
|  |  |  |  |  |  |  |  |
| DMSO-*d*_6_, 80°C [Hikichi et al., 1995] | | |  |  |  |  |  |
| 2,3,6-triacetylated | 4.77 | 4.67 | 5.15 | 3.80 | 3.90 | 4.38 | 4.14 |
| 2,3,6-triacetylated | 4.83 | 4.67 | 5.15 | 3.80 | 3.90 | 4.38 | 4.14 |
| 2,3,6-triacetylated | 4.94 | 4.78 | 5.18 | 3.94 | 3.77 | 4.32 | 4.21 |
| 2,3,6-triacetylated | 4.86 | 4.76 | 5.12 | 3.82 | 3.97 | 4.48 | 4.16 |
| 2,3-diacetylated | 4.78 | 4.63 | 5.07 | 4.83 | 3.55 | 3.79 | 3.69 |
| 2,3-diacetylated | 4.78 | 4.63 | 5.03 | 3.88 | 4.42 | 3.99 | 3.88 |
| 2,6-diacetylated | 4.67 | 4.59 | 3.68 | 3.60 | 3.75 | 4.38 | 4.14 |
| 3,6-diacetylated | 4.43 | 3.19 | 4.92 | 3.63 | 3.75 | 4.28 | 4.09 |
| 6-acetylated | 4.29 | 3.05 | 3.46 | 3.46 | 3.46 | 4.47 | 4.34 |
|  |  |  |  |  |  |  |  |
| DMSO-*d*_6_, 120°C [recent work, Knotek V., Ďurovič M., Dolenský B., Hrdlička Z., 2023] | | | | | | |  |
|  |  |  |  |  |  |  |  |
| 2,3,6-triacetylated | 4.682 | 4.599 | 5.058 | 3.740 | 3.770 | 4.312 | 4.085 |
| 2,3,6-triacetylated | 4.746 | 4.599 | 5.054 | 3.758 |  |  |  |
| 2,3-diacetylated | 4.618 | 4.564 | 4.986 | 3.774 | 3.445 | 3.727 | 3.629 |
| 3,6-diacetylated | 4.367 | 3.155 | 4.833 | 3.576 | 3.619 | 4.528 |  |

**References**

[Hikichi et al., 1995]

K. Hikichi, Y. Kakuta, T. Katoh: ^1^H NMR Study on Substituent Distribution of Cellulose Diacetate, *Polymer Journal* **1995**, 27 (7), 659–663.

[Kono et al., 2015]

H. Kono, H. Hashimoto, Y. Shimizu: NMR characterization of cellulose acetate: Chemical shift assignments, substituent effects, and chemical shift additivity, *Carbohydrate Polymers* **2015**, 118, 91–100.
