# Supplementary material for: Influence of Disinfection Methods on Cinematographic Film
Source: Materials (Basel). 2023 May 1;16(9):3493. doi: 10.3390/ma16093493 (PMC10180128; doi:10.3390/ma16093493)
Supplement: Supplementary file 1 [file materials-16-03493-s001.zip › Table S2.docx]

| **Table S2:** The chemical shifts of ^13^C NMR (ppm) relatively to TMS | | | | | |  |
| --- | --- | --- | --- | --- | --- | --- |
| DMSO-*d*_6_, 90°C [Kono et al., 2015] | |  |  |  |  |  |
| **Type of glucose unit** | **C1** | **C2** | **C3** | **C4** | **C5** | **C6** |
| 2,3,6-triacetylated | 98.8 | 71.3 | 72.0 | 75.7 | 71.7 | 61.8 |
| 2,3-diacetylated | 98.8 | 71.2 | 72.4 | 74.4 | 74.7 | 58.5 |
| 2,6-diacetylated | 99.0 | 72.9 | 71.6 | 79.6 | 71.6 | 62.3 |
| 3,6-diacetylated | 101.9 | 71.2 | 74.2 | 75.9 | 71.6 | 61.9 |
| 2-acetylated | 98.9 | 73.0 | 71.6 | 79.1 | 75.1 | 59.9 |
| 3-acetylated | 101.6 | 70.7 | 74.1 | 74.8 | 74.1 | 58.8 |
| 6-acetylated | 102.4 | 73.2 | 73.9 | 79.2 | 71.4 | 62.9 |
| unsubstituted | 102.0 | 72.9 | 73.9 | 79.1 | 74.3 | 60.1 |
|  |  |  |  |  |  |  |
| DMSO-*d*_6_, 120°C [recent work, Knotek V., Ďurovič M., Dolenský B., Hrdlička Z., 2023] | | | | | |  |
| 2,3,6-triacetylated | 98.721 | 71.343 | 72.047 | 75.274 | 71.684 | 61.764 |
| 2,3,6-triacetylated |  |  |  |  |  |  |
| 2,3-diacetylated |  |  | 72.470 | 74.290 | 74.760 | 58.490 |
| 3,6-diacetylated |  |  |  |  |  |  |

**References**

[Kono et al., 2015]

H. Kono, H. Hashimoto, Y. Shimizu: NMR characterization of cellulose acetate: Chemical shift assignments, substituent effects, and chemical shift additivity, *Carbohydrate Polymers* **2015**, 118, 91–100.
