# Supplementary figures and images for: Influence of Disinfection Methods on Cinematographic Film
Source: Materials (Basel). 2023 May 1;16(9):3493. doi: 10.3390/ma16093493 (PMC10180128; doi:10.3390/ma16093493)

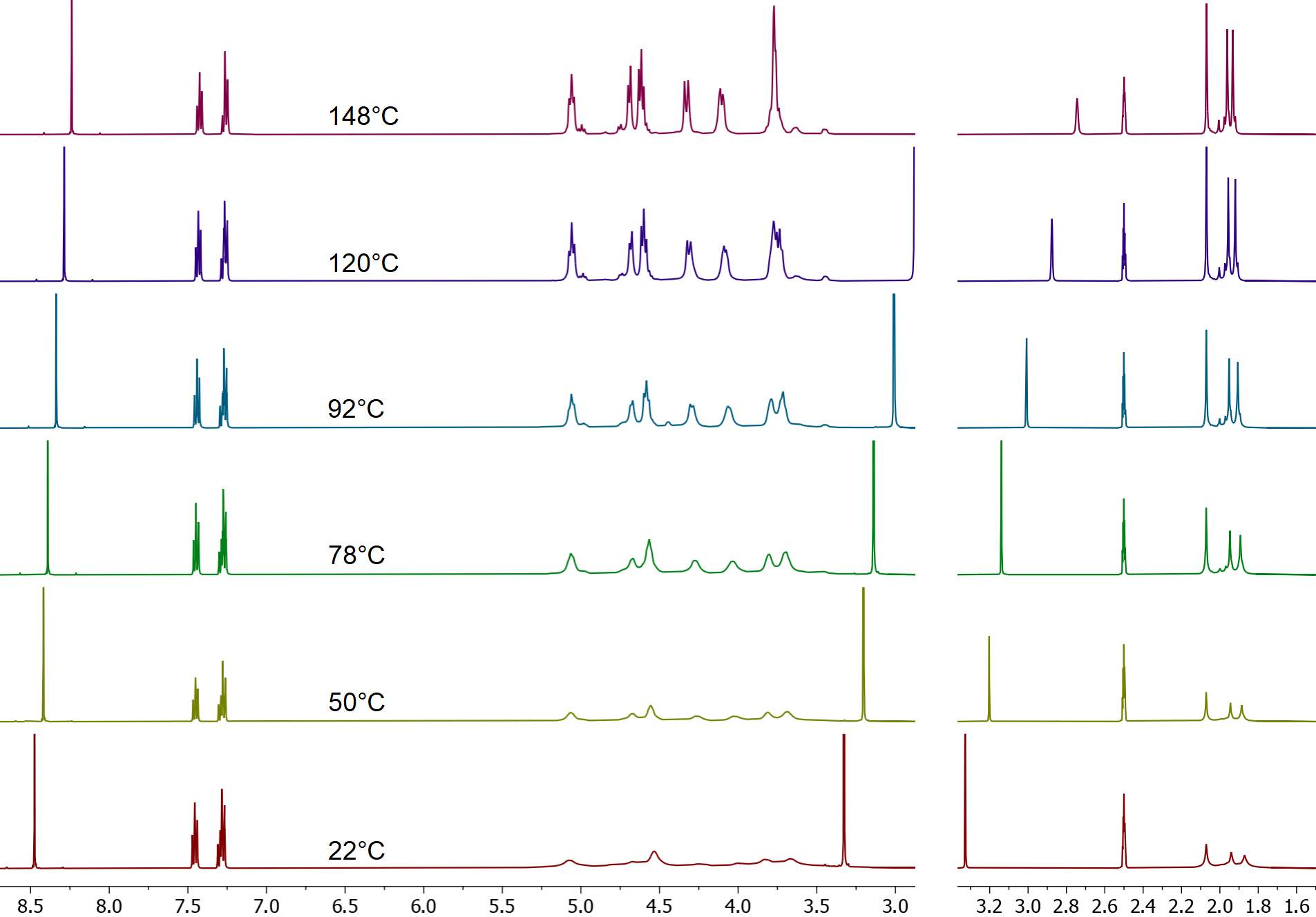

Supplement: Supplementary file 1 [file materials-16-03493-s001.zip › Figure S1.tif]

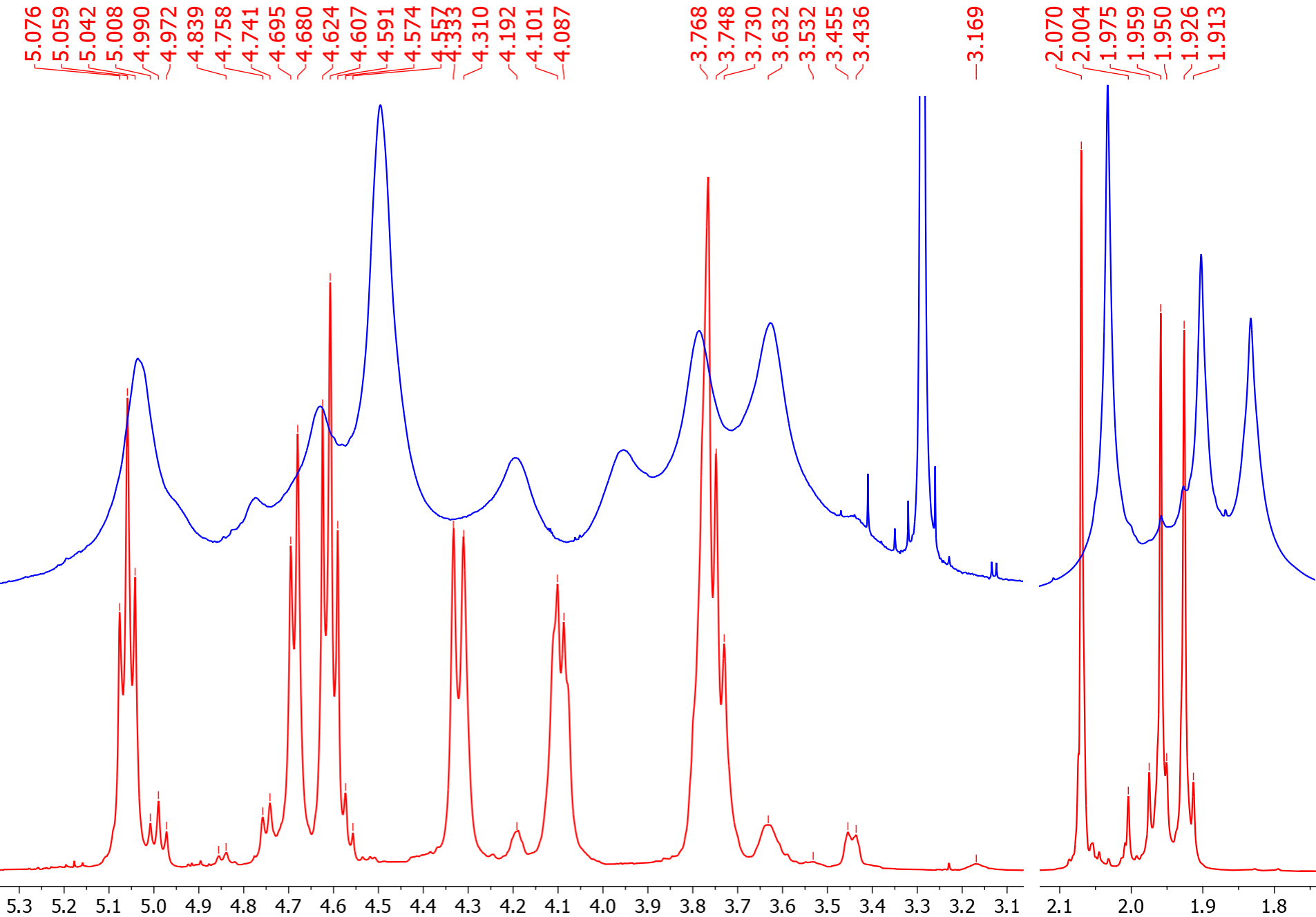

Supplement: Supplementary file 1 [file materials-16-03493-s001.zip › Figure S2.tif]

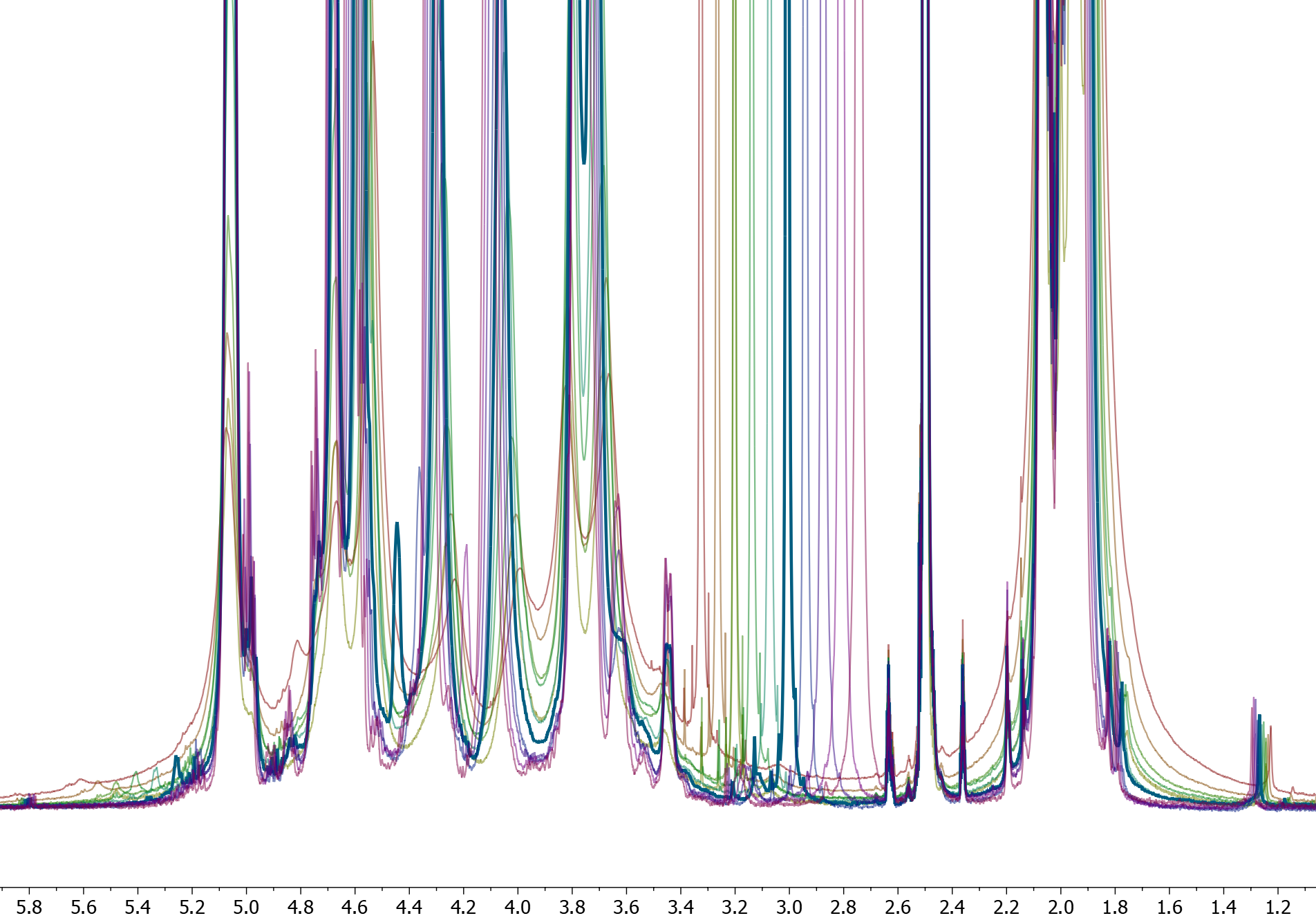

Supplement: Supplementary file 1 [file materials-16-03493-s001.zip › Figure S3.tif]

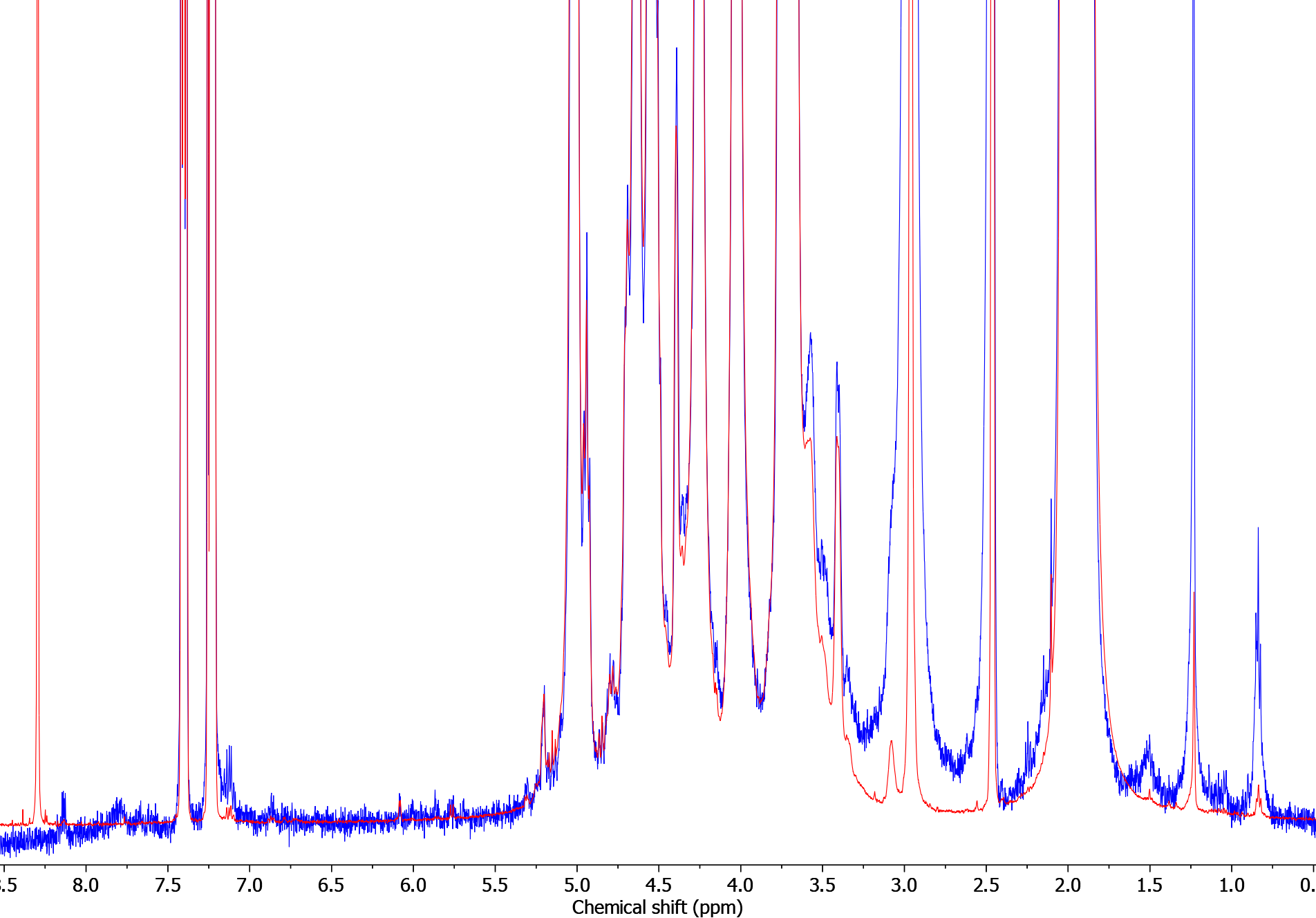

Supplement: Supplementary file 1 [file materials-16-03493-s001.zip › Figure S4.tif]

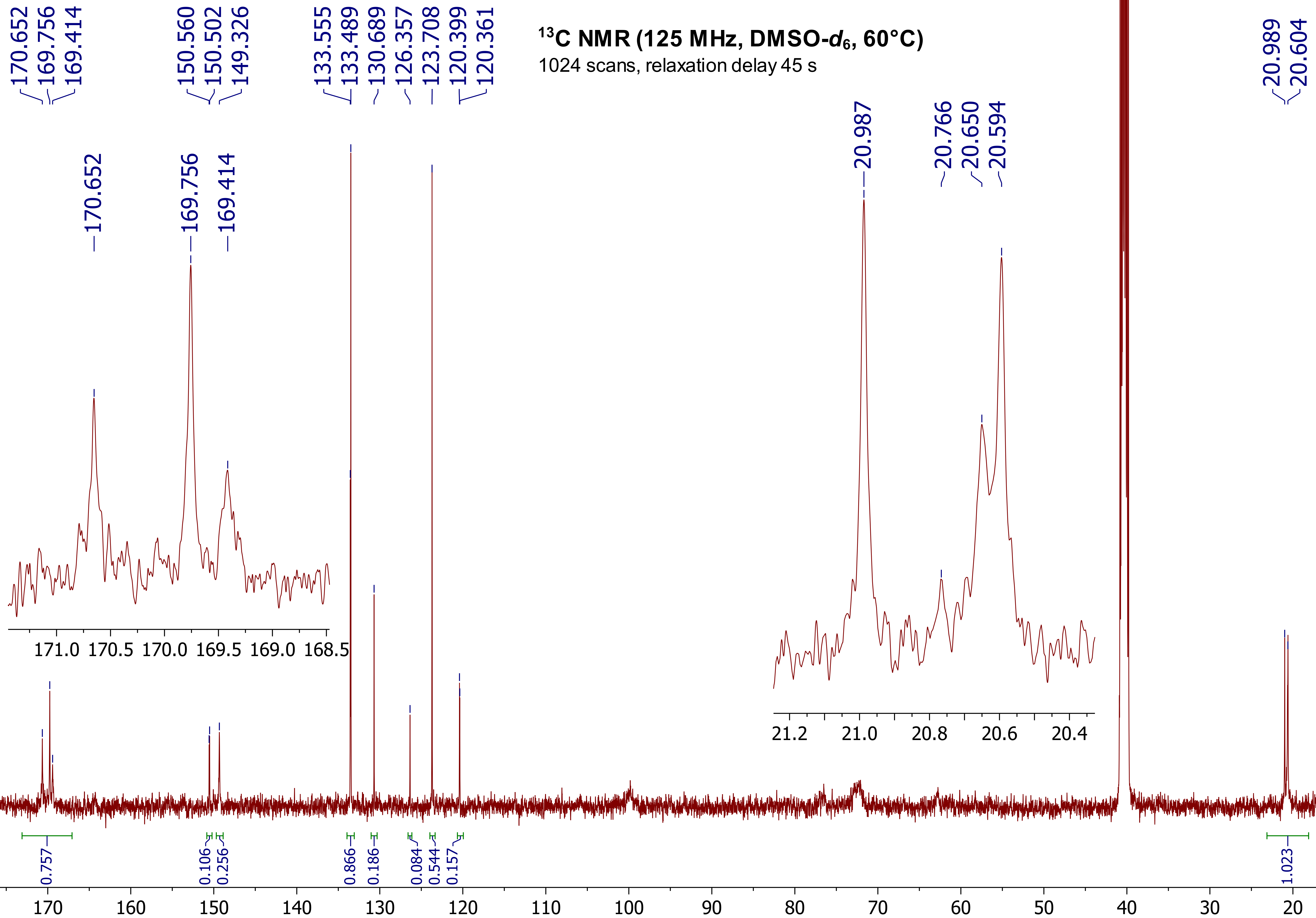

Supplement: Supplementary file 1 [file materials-16-03493-s001.zip › Figure S5.tif]
